# Poison by an Infusion of Poison Oak

**Published:** 1848-11

**Authors:** 


*Poisoning by an Infusion of Poison Oak.*—Mr. Wilkes, a student of medicine from Tennessee, curious to test the truth of the statement made by some writers on Materia Medica, that the *Rhus toxicodendron* only produces its poisonous effects when applied to the skin, tried a few weeks since, the experiment of drinking a strong decoction of the plant. He boiled the vine, with its leaves, and drank about a gill of the fluid, taking care, while preparing it, not to let the vine come in contact with his person. It was taken after supper, and next morning he found his face much swollen. The swelling continued to increase until his eyes were completely closed. He resorted at once to a wash, composed of perchloride of mercury gr. i., sal ammoniac grs. ii., water ℥ ij, which he had prepared in the event of his being poisoned. In about thirty-six hours the swelling and inflammation had disappeared. He slept nearly the whole time his eyes were closed, showing the narcotic action of the article.—*Western Journal of Med. and Surg.*

